# Southern Europe’s Coming Plagues: Vector-Borne Neglected Tropical Diseases

**DOI:** 10.1371/journal.pntd.0004243

**Published:** 2016-06-30

**Authors:** Peter J. Hotez

**Affiliations:** 1 Departments of Pediatrics and Molecular Virology and Microbiology, National School of Tropical Medicine, Baylor College of Medicine, Houston, Texas, United States of America; 2 Sabin Vaccine Institute and Texas Children’s Hospital Center for Vaccine Development, Houston, Texas, United States of America; 3 Department of Biology, Baylor University, Waco, Texas, United States of America; 4 James A. Baker III Institute for Public Policy, Rice University, Houston, Texas, United States of America; 5 Scowcroft Institute of International Affairs, The Bush School of Government and Public Service, Texas A&M University, College Station, Texas, United States of America; Yale School of Public Health, UNITED STATES

New social and environmental forces, including economic downturns, climate change, and human migrations from the Middle East and North Africa, are merging into a “perfect storm” to promote the widespread emergence of neglected tropical diseases (NTDs) in Southern Europe.

**Fig 1:**
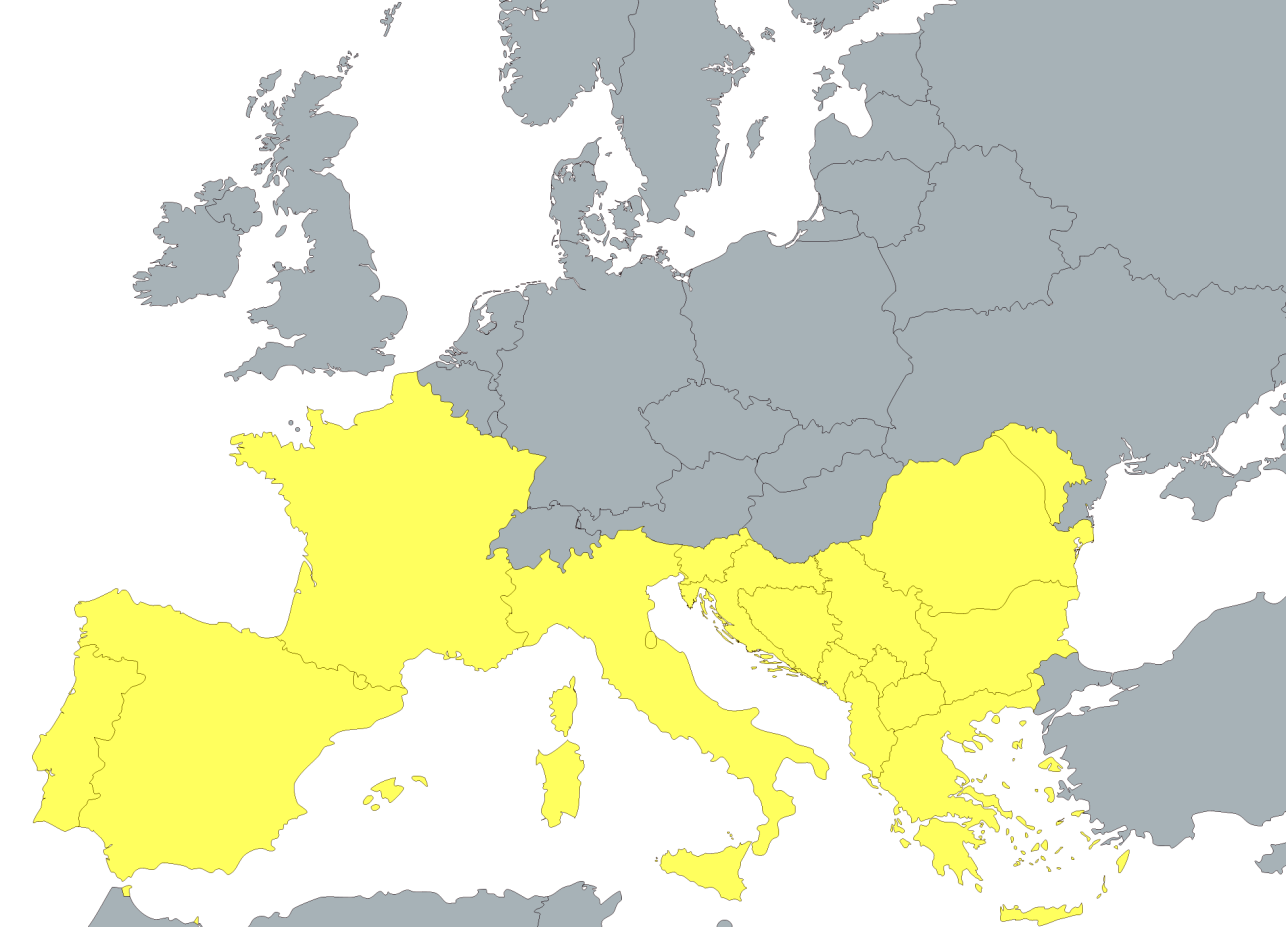
Southern Europe. Map of Southern Europe. Figure created with mapchart.net

There is no single definition of the area or countries comprising Southern Europe, but most focus on the countries aligning the Mediterranean Sea, including Spain and Portugal (comprising the Iberian Peninsula), Italy (especially the portion on the Italian Peninsula), Southern France and Corsica, and Greece (Fig 1). The Balkan countries in southeastern Europe, including Croatia, are also sometimes included in this definition.

Throughout recorded history, vector-borne tropical infections have flourished in the warm Mediterranean climate of Southern Europe. For centuries, malaria (known as Roman estivo-autumnal fever) was endemic to the Pontine Marshes and low-lying area known as the Campagna surrounding Rome [1]. Some scholars have even attributed the decline of the Roman Empire partly to malaria [2,3], while others have conversely linked the dramatic upturns in the economies of Southern European countries to malaria elimination in the years following World War II [4]. Similarly, leishmaniasis caused by *Leishmania infantum* has also been an important vector-borne NTD in Southern Europe, as well as an important opportunistic infection in the region’s HIV/AIDS epidemic [5,6].

Over the last decade, however, a number of vector-borne diseases have emerged or re-emerged in Southern Europe. The major diseases are highlighted in Box 1, and include five diseases listed among WHO’s 17 NTDs, including Dengue, Chagas disease, leishmaniasis, opisthorchiasis, and schistosomiasis, as well as five others listed within the scope of *PLOS Neglected Tropical Diseases*—other arbovirus infections and vivax malaria—in addition to the borrelioses (relapsing fever is also in the scope of *PLOS Neglected Tropical Diseases*).

**Box 1. Emerging Vector-Borne Diseases and Neglected Tropical Diseases in Southern Europe**

**Table:** 

Viral Infections	Chikungunya
	Dengue (DENV-1)
	West Nile virus infection
	Crimean-Congo hemorrhagic fever
	Toscano virus infection
Bacterial Infections	Lyme Disease and other borrelioses
Protozoan Infections	Chagas disease
	Leishmaniasis
	Vivax malaria
Helminth Infections	Opisthorchiasis
	Schistosomiasis

Of particular concern is the recent emergence or re-emergence of arbovirus infections [7]. In terms of indigenous transmission, Chikungunya first emerged in Italy in 2007, with a second outbreak of in France in 2010 [7–10]. According to WHO, chikungunya also emerged in Spain in 2015 [11]. In 2012–2013, dengue fever re-emerged (after a period of decades) on Madeira off of the Atlantic coast of Portugal [12–14]. More than 1,000 cases were confirmed in the outbreak, caused by dengue virus type 1 (DENV-1), which may have been introduced from tropical regions of South America [12–14]. In Europe, both DENV-1 and Chikungunya are transmitted by *Aedes albopictus* (the Asian tiger mosquito) [15]. *Ae*. *albopictus* was first detected in Spain in 2004 [16].

West Nile virus (WNV) infection, transmitted by Culex mosquitoes, has also emerged in Southern Europe [17,18]. According to the European Centre for Disease Control and Prevention (ECDC), WNV re-emerged in Romania in 1996, but today the nation of Greece has the highest case rate, with Athens also being affected [7]. WNV cases are also occurring in southeastern Europe—Bulgaria, Hungary, and Romania—as well as in Italy [7], and there are concerns that these Southern European foci could spread to other parts of Europe [19].

Certain vector-borne viruses are endemic to Southern Europe. For instance, Toscano virus, transmitted by sand flies, is a significant cause of disease and aseptic meningitis in Italy [6]. Among the tick-borne arboviruses, Crimean-Congo hemorrhagic fever (CCHF) is endemic to the Balkans, with Turkey exhibiting the highest numbers of cases [7], but the infection has also emerged in Greece [20]. In contrast to CCHF, the flavivirus responsible for tick-borne encephalitis is mostly found in northeastern Europe [7], while the highest endemicity of Lyme borreliosis, a bacterial vector-borne disease, may be in Central Europe [21].

Vector-borne parasitic infections are also now important NTDs emerging in Southern Europe. Chagas disease is increasingly reported in Spain and elsewhere in Southern Europe due to importation from Bolivia where the prevalence rate is the highest, as well as Argentina, Brazil, and Mexico where the largest numbers of people are currently living with Chagas disease [22,23]. However, in terms of autochthonous transmission, leishmaniasis, caused mostly by *Leishmania infantum* and transmitted by *Phlebotomus* sandflies, has emerged as the most important protozoan NTD, especially as an opportunistic infection in AIDS patients [24,25]. *L*. *infantum* is a zoonotic infection that relies on canine reservoir hosts, and results in both human cutaneous and visceral leishmaniasis. *Leishmania tropica*, the major cause of old world anthroponotic cutaneous leishmaniasis in the Middle East and North Africa, has also occurred sporadically in Greece and elsewhere in Southern Europe [24].

Greece underwent a national malaria elimination effort in the years following World War II and up to 1960s [26]. As noted above, such efforts are believed to have promoted important economic returns on investment [4]. However, in 2009, autochthonous cases of vivax malaria were reported regularly from Greece, and have continued annually ever since [7,27]. Thus, these cases in Greece represent the first consistent European indigenous transmission of malaria in many years [27]. A new geospatial analysis has now identified transmission hotspots in Greece where it is expected that indigenous malaria transmission will continue [26]. Autochthonous malaria transmission has also been reported from Italy and Spain [7,28,29].

In terms of helminth infections, there is evidence for the Southern European emergence of snail-transmitted fluke infections, including opisthrochiasis in Italy [30], and schistosomiasis in Corsica, off the coast of southern France [31].

With the exceptions of CCHF and Lyme borreliosis, Southern Europe has become “ground zero” for the vector-borne NTDs now significantly affecting the public health and, potentially, the future economy of the Eurozone. Many of these diseases, such as chikungunya, dengue fever, WNV, malaria, and the snail-transmitted helminth infections, have emerged or re-emerged only in the past few years. Indeed, there is now a startling similarity between the vector-borne diseases patterns of North Africa and Southern Europe so that all the nations surrounding the Mediterranean Sea have become almost unified with respect to their endemic NTDs.

The factors responsible for promoting the vector-borne NTDs in Southern Europe are under investigation, but there are some key lead possibilities to consider.

- *Poverty*. Throughout the world’s low- and middle-income countries, poverty is a major social determinant promoting the ongoing transmission of NTDs. Previous findings have determined that comparable levels of extreme poverty can also be found among the G20 countries and are also contributing to widespread NTDs [32,33]. It is interesting to note how the emergence or re-emergence of Southern Europe’s major NTDs roughly coincides with the European debt crisis that began in 2009 when countries such as Greece, Portugal, and Spain experienced difficulties in repaying their government debts without outside assistance. Ultimately, Greece defaulted on its debt to the International Monetary Fund in 2015, thereby precipitating a financial crisis linked to high unemployment. There is an important need to better understand the link between poverty and NTDs. So far, it has been found that NTDs flourish in impoverished settings, but also that NTDs exhibit a unique ability to reinforce poverty through their debilitating effects on workers, women, and growing and developing children.
- *Mass human migrations*. Still another key social factor may be the humanitarian crisis linked to hundreds of thousands of people fleeing conflicts in Libya, Syria, and Iraq due to the occupation of ISIS [34]. In so doing, they could be introducing or re-introducing NTDs endemic to the Middle East and North Africa, including the vector-borne NTDs highlighted above. For example, cutaneous leishmaniasis in Syria, where it is often known as “Aleppo Evil,” has reached hyperendemic proportions due to breakdowns in health systems and lack of access to essential medicine, with at least tens of thousands of new cases annually [35]. Quite possibly both cutaneous leishmaniasis and sand fly vectors are being routinely re-introduced into Southern Europe.
- *Climate change*. Finally, it has been noted that outside of the Arctic region, Europe’s single largest temperature increases associated with serious heat waves are now occurring in Southern Europe [36]. The factors promoting climate change include increased greenhouse gas emissions as a result of agriculture; burning of coal, oil, and gas (fossil fuels); landfills; and industrial pollutants [36]. Increased temperatures are helping to facilitate the survival and longevity of insects and snails with the capacity to transmit NTDs. Climate change may also promote the spread of some of Southern Europe’s vector-borne NTDs to Northern Europe, including WNV and leishmaniasis [19,37].

The needs for tackling Southern Europe’s emerging NTDs are pervasive and will need to include increased active surveillance activities, studies to elucidate the modes of indigenous transmission, and prevention measures. The ECDC has helped to establish an innovative European Environment and Epidemiology (E3) Network that will include geospatial mapping for next generation emerging vector-borne disease threats [26]. In parallel, many of the tools needed to control or eliminate these newly emerging NTDs have not yet been developed and we will require an aggressive program of focused research and development (R&D) on this front. Previously (together with a colleague), I have proposed the establishment of such a R&D center in Greece for this purpose [38]. Our rationale in proposing to locate the institute in Greece is because this country is geographically located at the confluence of the three major forces highlighted above—European poverty, the refugee crisis, and climate change—and because of their existing strengths in biotechnology. There is also an urgency to maintain biotechnology infrastructure in Greece (and prevent further brain drain) in light of its recent economic downturns and hardships. In addition, however, we must now consider dedicated funds from the European Union or perhaps another European body in order to launch initiatives to combat emerging NTDs and other vector-borne diseases in Southern Europe.
